# Genome-Wide Analysis of the Indispensable Role of Non-structural Proteins in the Replication of SARS-CoV-2

**DOI:** 10.3389/fmicb.2022.907422

**Published:** 2022-06-01

**Authors:** Yunyun Jin, Muzi Ouyang, Ting Yu, Jiaxin Zhuang, Wenhao Wang, Xue Liu, Fangfang Duan, Deyin Guo, Xiaoxue Peng, Ji-An Pan

**Affiliations:** The Center for Infection and Immunity Study and Molecular Cancer Research Center, School of Medicine, Sun Yat-sen University, Shenzhen, China

**Keywords:** SARS-CoV-2, nsp, viral replication and transcription, indispensable role, replicon

## Abstract

Understanding the process of replication and transcription of SARS-CoV-2 is essential for antiviral strategy development. The replicase polyprotein is indispensable for viral replication. However, whether all nsps derived from the replicase polyprotein of SARS-CoV-2 are indispensable is not fully understood. In this study, we utilized the SARS-CoV-2 replicon as the system to investigate the role of each nsp in viral replication. We found that except for nsp16, all the nsp deletions drastically impair the replication of the replicon, and nsp14 could recover the replication deficiency caused by its deletion in the viral replicon. Due to the unsuccessful expressions of nsp1, nsp3, and nsp16, we could not draw a conclusion about their *in trans*-rescue functions. Our study provided a new angle to understand the role of each nsp in viral replication and transcription, helping the evaluation of nsps as the target for antiviral drug development.

## Introduction

The ongoing pandemic of COVID-19 caused by severe acute respiratory syndrome coronavirus 2 (SARS-CoV-2) has posed a serious threat to human health and led to heavy economic loss (V’Kovski et al., 2021). The relatively limited knowledge of this deadly virus hinders us from efficiently treating patients with COVID-19 (Feng et al., 2020). Besides the recognition and entry process (Lan et al., 2020; Yin et al., 2022), the viral replication and transcription process regulated by viral replicase proteins possesses many promising targets for antiviral strategy development (V’Kovski et al., 2021; Malone et al., 2022). Thus, the systemical analysis on the roles of proteins derived from viral replicase is an important biomedical objective.

SARS-CoV-2, belonging to the *Nidovirales* order, the *Coronaviridae* family, and the *Betacoronavirus* genus (Coronaviridae Study Group of the International Committee on Taxonomy of Viruses, 2020), is an enveloped positive-strand RNA virus. Its genome contains at least nine open reading frames (ORFs). The 5′-terminal two-thirds of the viral genome contains two open reading frames, ORF1a and ORF1ab (Chen et al., 2020). The latter is translated by a -1 ribosomal frameshifting mechanism, in which the translational complex avoids the stop codon by altering the reading frame while encountering the “slippery” sequence at the terminus of ORF1a (Pan et al., 2008). The translational products of ORF1a and ORF1ab, polyprotein 1a (pp1a) and polyprotein 1ab (pp1ab), are responsible for the viral replication and transcription. Before going to its final roles, pp1a and pp1ab are processed up to 16 non-structural proteins (nsps) by their own proteases, papain-like protease (PLpro) located in nsp3 and 3C-like protease (3CLpro) or main protease (Mpro) of nsp5 (Pan et al., 2008; Du et al., 2021; Jiang et al., 2021).

Nsp1 (approximately 180 aa in SARS-CoV-2) is a multifunctional protein capable of altering host translation (Narayanan et al., 2008a; Kamitani et al., 2009; Lokugamage et al., 2012), triggering host mRNA cleavage (Kamitani et al., 2009; Huang et al., 2011) and decay (Kamitani et al., 2006; Narayanan et al., 2008b), inhibiting the innate immune response (Zust et al., 2007; Narayanan et al., 2008b), and inducing cytokines and chemokines (Law et al., 2007). By introducing deletions in murine hepatitis viruses (MHV) and the analysis of mutant MHV, another member of the *Betacoronavirus* genus, Brockway and Denison (2005) identified a few residues in nsp1 important for viral RNA synthesis and replication, and viral protein processing. The SARS-CoV-2 nsp1 is an evolving protein, as, besides mutations, two deletions in nsp1 were identified, including the deletion of 686–694 nt (Benedetti et al., 2020) and the deletion of 500–532 nt (Lin et al., 2021b).

Nsp2’s (approximately 638 aa in SARS-CoV-2) function is not well defined, while it may be involved in regulating host intracellular signaling through the interaction with prohibitin 1 (PHB1) and PHB2 (Cornillez-Ty et al., 2009). SARS-CoV-2 with the variation of nsp2 (T85I) may lead to poor replication in Vero-CCL81 cells (Pohl et al., 2021). Furthermore, the genetic deletions of nsp2 on the reverse genetics systems of MHV and SARS-CoV lead to attenuated viral growth and RNA synthesis (Graham et al., 2005).

Nsp3 (approximately 1945 aa in SARS-CoV-2) is a large multi-domain protein. It encompasses multiple functional domains, including ubiquitin-like domain, single-stranded poly(A) binding domain, C-terminal SARS-Unique domain, PLpro domain, nucleic acid-binding domain, and two transmembrane helix motifs (Jiang et al., 2021). The PLpro activity of nsp3 is responsible for releasing nsp1, nsp2, and nsp3 *per se* from the pp1a or pp1ab. Thus, it is recognized as a promising target for antiviral drug development (Shin et al., 2020). Besides protease activity, nsp3 has deubiquitinating and interferon antagonism activities (Clementz et al., 2010) and may regulate the viral replication by interacting with viral nsps, including nsp1, nsp4, nsp6, nsp10, nsp12, nsp13, nsp14, and N (Baez-Santos et al., 2015; Jiang et al., 2021). Nsp3 could also benefit viral replication by improving the inter/intra-cellular microenvironment. SARS-CoV-2 nsp3 delays the expression of IFN-β (Lei et al., 2020).

Nsp4 (approximately 500 aa in SARS-CoV-2) also has multiple transmembrane domains. Nsp4 of SARS-CoV interacts with nsp3, contributing to the endoplasmic reticulum (ER) membrane rearrangement and the assembly of double-membrane vesicles (DMVs), which play an essential role in viral replication and transcription (Sakai et al., 2017). The essential role of nsp4 was also supported by the sequence analysis on patient samples, and the nsp4 variant (E3073A) of SARS-CoV-2 is associated with a significantly reduced fever duration (Zekri et al., 2021).

Nsp5 (approximately 306 aa in SARS-CoV-2) is one of the most widely studied proteins of SARS-CoV-2. Nsp5 plays a crucial role in the maturation of viral replicase polyprotein by cleaving pp1ab at 11 sites and subsequently releasing nsp4 to nsp16 for the assembly of replication and transcription complex (RTC) (Jin et al., 2020). Nsp5 is recognized as a promising target for antiviral drug development, and more than 86 potential inhibitors of nsp5 were selected by various studies (Yan and Gao, 2021). Its structures with or without inhibitors are resolved by a myriad of studies (Mariano et al., 2020).

Nsp6 (approximately 290 aa in SARS-CoV-2) also has transmembrane domains. Together with nsp3 and nsp4, nsp6 contributes to the formation of DMV (Angelini et al., 2013). Different from the function of nsp3-nsp4 complex in pairing membranes, nsp6 majors in membrane proliferation. Nsp6 was shown to limit autophagosome expansion (Cottam et al., 2014).

Nsp7 (approximately 83 aa in SARS-CoV-2) and nsp8 (approximately 198 aa in SARS-CoV-2) function as the cofactors for RTC (Kirchdoerfer and Ward, 2019). The involvement of nsp7 and nsp8 in RTC goes through a transition from nsp7–nsp8 hexadecameric primase complex to the nsp12–nsp7–nsp8 polymerase complex, promoting RdRP efficiency of viral RNA product synthesis (Wang et al., 2020).

Nsp9 (approximately 113 aa in SARS-CoV-2) plays a vital role in the replication of SARS-CoV-2 through its activity in ssRNA/DNA binding ability (Littler et al., 2020), which is regulated by its dimerization (de et al., 2021) and NMPylation on its conserved site (Slanina et al., 2021). SARS-CoV-2 nsp9 stimulates type I interferon response (Lei et al., 2020).

Nsp10 (approximately 139 aa in SARS-CoV-2) was identified as an interaction partner of nsp14 and nsp16 by the genome-wide screening of intraviral protein–protein interactions (Pan et al., 2008). Nsp10 interacts with nsp14 (Lin et al., 2021a) and nsp16 (Krafcikova et al., 2020) to promote their 3’–5’ exonuclease and RNA ribose-2’-O-methylation activities, respectively.

Nsp11 (approximately 13 aa in SARS-CoV-2) is a cleavage product of pp1a processed by Mpro at the nsp10/11 boundary. Nsp11 shares the same first nine amino acids with nps12 and exhibits an intrinsically disordered protein behavior (Gadhave et al., 2021).

Nsp12 (approximately 932 aa in SARS-CoV-2), the RNA-dependent RNA polymerase (RdRP) of SARS-CoV-2, forms the RTC with nsp7 and nsp8 (Wang et al., 2020). Mutations, S759A/D760A/D761A, at the key residues in nsp12 diminished the viral replication (Jin et al., 2021). More than 95% identical to SARS-CoV counterpart, nsp12 of SARS-CoV-2 exhibits a similar sensitivity to the inhibitory effect of remdesivir, and the decreased enzymatic activity and thermostability (Peng et al., 2020). Besides RdRP activity, SARS-CoV-2 nsp12 is responsible for viral RNA capping as a GTPase, adding a GTP nucleotide to the 5’ end of viral RNA via a 5’–5’ triphosphate linkage (Walker et al., 2021).

Nsp13 (approximately 601 aa in SARS-CoV-2) possesses RNA helicase and the nucleoside triphosphate hydrolase (NTPase) activities, unwinding viral RNA duplex and supplying the energy for unwinding by hydrolyzing ATP, respectively, in the replication of viral RNAs (Shu et al., 2020). Thus, RNA helicase activity of nsp13 is sensitive to the concentration of ATP. The increased ATP concentrations promote the processivity of nsp13 in unwinding duplex RNA (Jang et al., 2020). Two mutations, Y541C and P504L, from variants of SARS-CoV-2, located in the nucleotide-binding core of nsp13, weaken the transmission capacity of SARS-CoV-2, indicating the crucial role of nsp13 in viral replication (Wang et al., 2021).

Nsp14 (approximately 527 aa in SARS-CoV-2) has 3’–5’ exoribonuclease (ExoN) and N7-guanine methyltransferase (N7-MTase) activities, which are born by N-terminal and C-terminal domains, respectively (Chen et al., 2009). The nsp14 ExoN activity is a key component of the RNA proofreading machinery, which is proposed to be essential for the stability and replication efficiency of viral genome (Becares et al., 2016). Nsp10 interacts with the N-terminal domain of nsp14, promoting ExoN activity (Ma et al., 2015). Its N7-MTase activity plays a crucial role in the synthesis of viral mRNA cap, preventing the recognition by the host cell (Becares et al., 2016). Both enzymatic activities of nsp14 are crucial for viral replication, and the mutations of D90A/E92A and D331A, impairing the ExoN and N7-MTase, respectively, drastically decreased the generation of viral genomic/subgenomic RNAs in viral replicon system (Jin et al., 2021).

Nsp15 (approximately 346 aa in SARS-CoV-2) is a uridine-specific endoribonuclease (EndoU) whose activity resides in its C-terminal domain (Frazier et al., 2021). Nsp15 plays a crucial role in viral replication, because the inactivation of NendoU by introducing mutations in nsp15 and the deletion in nsp15 drastically decreased the viral replication and recovery (Ivanov et al., 2004; Almazan et al., 2006; Kang et al., 2007; Nguyen et al., 2021).

Nsp16 (approximately 298 aa in SARS-CoV-2) mediates 2’-O-methylation of viral RNA cap structure, preventing the degradation by host nucleases (Krafcikova et al., 2020; Wilamowski et al., 2021). To perform its MTase activity, nsp16 requires nsp10 as a stimulatory factor to bind its m7GpppA-RNA substrate and S-adenosyl-L-methionine (SAM), methyl donor (Chen et al., 2011). In contrast, nsp10 also interacts with nsp14, another MTase of SARS-CoV-2, but is not required for MTase activity of nsp14 (Ma et al., 2015). SARS nsp16 plays a crucial role in the viral replication, because the elimination of the nsp16 expression by introducing the stop codon at its 5’ end largely attenuated the viral replication (Almazan et al., 2006; Rohaim et al., 2021).

As summarized above, the functional characterization of nsps indicates their potential involvement in viral replication and transcription. A number of studies showed that the overexpression of some nsps could further promote the replication level of viral reverse genetics systems, indicating that these nsps play essential roles in viral replication but possibly are not sufficient in the viral replication complex, which is composed of the nsps processed from replicase polyprotein (Jin et al., 2021; Luo et al., 2021).

In this study, to clarify whether each nsp is indispensable for viral replication and transcription, we examined the replication of the viral replicon with the deletion of each nsp. To minimize the undesired impact of each nsp deletion on the viral polyprotein process, we reconstructed cleavage sites recognized by PLpro and 3CLpro and confirmed the cleavage efficiency using our PLpro/3CLpro activity reporter system. By monitoring the replication activity of viral replicons with each nsp deletion, we found that the dependencies of viral replication on each nsp varied considerably, and nsp14 can rescue the decreased replication of viral replicon caused by the loss of nsp14 *per se*.

## Results

### The Reconstituted Cleavage Sites Can Be Efficiently Cleaved by Papain-Like Protease and 3C-Like Protease

**FIGURE 1:**
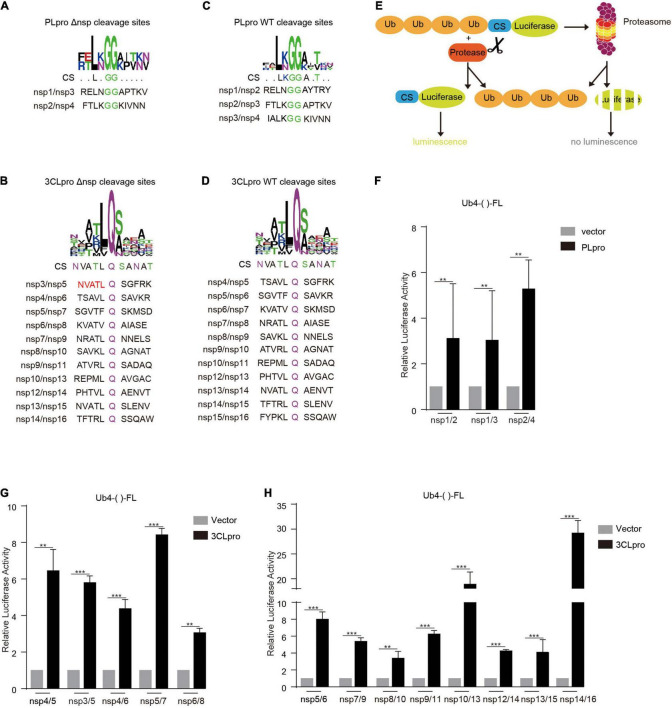
Reconstituted cleavage sites are cleaved by PLpro and 3CLpro. The reconstituted **(A,B)** and WT **(C,D)** cleavage site sequences were analyzed using WEBLOGO. Note that the reconstituted and WT cleavage sites have similar consensus sequences (CSs). **(E)** Schematic illustration of PLpro and 3CLpro activity reporter system. Four tandem ubiquitins mediate the degradation of luciferase through the proteasome pathway. The cleavage site of PLpro or 3CLpro is inserted between the four tandem ubiquitins and the luciferase. PLpro or 3CLpro recognizes and cuts the cleavage site, leading to the detachment of the luciferase from the four tandem ubiquitins. The luciferase activity is measured and reflects the PLpro or 3CLpro activity. HEK293T cells were transfected with vector or PLpro **(F)** and 3CLpro **(G,H)**, pRL-TK, and Ub4-FL inserted with indicated various cleavage sequences (Ub4-()-FL). 24 h post-transfection, the cells were collected, and the lysates were subjected to Dual-Luciferase Reporter (DLR™) Assay. The data represent one of three independent experiments with similar results; error bars represent the mean ± s.e.m. Two-tailed unpaired Student’s *t*-test or one-way ANOVA with Bonferroni post-test correction was used to analyze the significance; **P < 0.01 and ***P < 0.001; ns, not significant.

The deletion of nspx (x is any number from 1 to 16) from the viral genome can lead to the failure to separate its adjacent upstream (nspx-1) and downstream nsps (nspx + 1) (Supplementary Figure 1). The functions of nspx-1 and nspx + 1 are likely altered in the fused form, nspx-1-nspx + 1. To solve this issue, we reconstituted the cleavage sites between nspx-1 and nspx + 1, adjacent to the removed nspx. The reconstituted cleavage sites are composed of the C-terminal amino acid sequence of nspx-1, double glycine (GG) or glutamine (Q), and N-terminal amino acid sequence of nspx + 1 (Figures 1A,B). The consensus sequence of reconstituted cleavage sites is similar to that of the wild-type cleavage sites (Figures 1C,D). For nsp3/nsp5 cleavage site, we replaced the last five amino acids, IALKG, at the C-terminal end of nsp3 with NVATL to rebuild the cleavage site recognized by 3CLpro (Figure 1B).

To verify whether the reconstituted cleavage sites could be processed by PLpro and 3CLpro, we employed the 3CLpro activity reporter system developed previously by our group (Du et al., 2021; Figure 1E). In this system, the reporter gene firefly luciferase (FL) is fused with four tandem ubiquitins (Ub4), which lead to the degradation of firefly luciferase in a ubiquitin-dependent proteasome mechanism and low activity of firefly luciferase. An in-frame amino acid sequence is inserted at the *Hin*dIII site between four tandem ubiquitins and firefly luciferase (Ub4-()-FL). The cleavage on this inserted sequence leads to the separation of four tandem ubiquitins and firefly luciferase, which gives a robust increase in the detected activity of firefly luciferase.

We inserted all the reconstituted cleavage site sequences at the *Hin*dIII site and tested the cleavage efficiency by PLpro and 3CLpro. The results showed that the cleavage efficiencies on the reconstituted cleavage sites are comparable to that of WT cleavage sites, indicating that the deletion of nsps, except nsp3 with PLpro activity and nsp5 with 3CLpro activity, should not influence the process of replicase polyprotein (Figures 1F–H).

### Construction of pBAC-nCoV-Replicon-Δnsp (nCoV-Rep-Δnsp)

**FIGURE 2:**
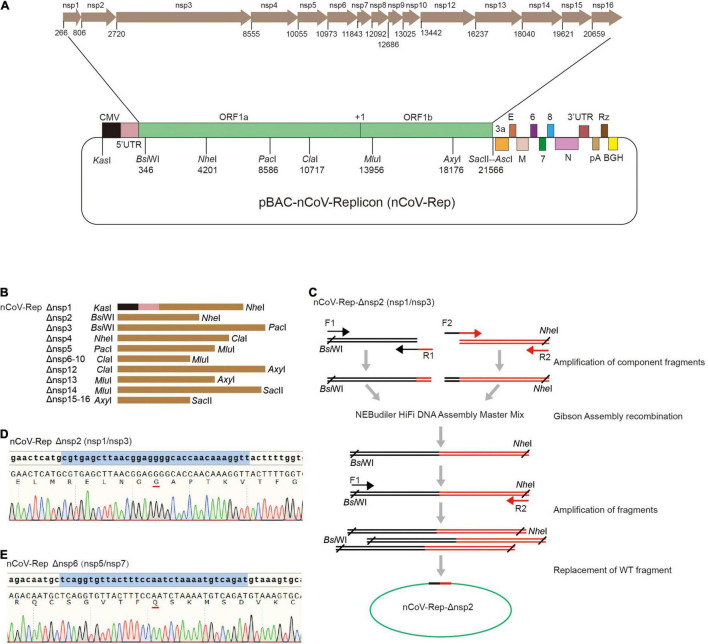
Construction of nCoV-Rep-Δnsp. **(A)** Schematic of the SARS-CoV-2 replicon (pBAC-nCoV-Replicon) with indicated unique restriction sites and the location of each nsp. **(B)** The design of the fragments with indicated restriction sites for the deletion of each nsp. **(C)** Schematic of the construction strategy for cloning nCoV-Rep-Δnsp plasmids. The Sanger sequencing results of reconstituted sequences in nCoV-Rep-Δnsp2 **(D)** and nCoV-Rep-Δnsp6 **(E)**.

Next, we constructed the replicon of SARS-CoV-2 with the deletions of nsp1 to nsp16 based on pBAC-nCoV-Replicon (nCoV-Rep), which was constructed by our group previously (Jin et al., 2021). In CMV-5’ UTR-ORF1ab region of nCoV-Rep, we designed eight unique restriction sites, namely, *Kas*I, *Bsi*WI, *Nhe*I, *Pac*I, *Cla*I, *Mlu*I, *Axy*I, and *Sac*II, which separate the viral cDNA sequence into seven replaceable segments and thus are very helpful for the reconstruction operation on the replicon (Figure 2A). For the deletion of each nsp, we first selected the segment containing the target nsp with the unique restriction sites. The segments for various nsps are described in Figure 2B.

To obtain the target nsp-deleted segment, we designed middle forward (F2) and reverse (R1) primers composed of 3’ terminal sequence of the upstream nspx-1 adjacent to the deleted nspx, cleavage site sequences, encoding GG or Q, and 5’ terminal sequence of the downstream nspx + 1 adjacent to the deleted nspx (Figure 2C), except the primers for nsp4 deletion, which contain the mutant sequence encoding NVATL (Figure 1B). We used the primer pair of the forward primer (F1) upstream of the restriction site and the middle reverse primer (R1) and the primer pair of the reverse primer (R2) downstream of restriction site and the middle forward primer (F2) to amplify two-component fragments. We assembled two-component fragments into the segment with deletion of the target nsp using Gibson Assembly strategy and amplified the segment with the primer pair of the forward primer upstream (F1) of the restriction site and the reverse primer (R2) downstream of the restriction site (Figure 2C). Using the unique restriction sites, we replaced WT segments with the target nsp-deleted segments and verified the segment sequence with Sanger DNA sequencing to ensure no undesired mutations (Figures 2D,E and Supplementary Figure 2).

### The Deletion of Non-structural Protein 1–15 Impairs the Replicative Activity of the Replicon

**FIGURE 3:**
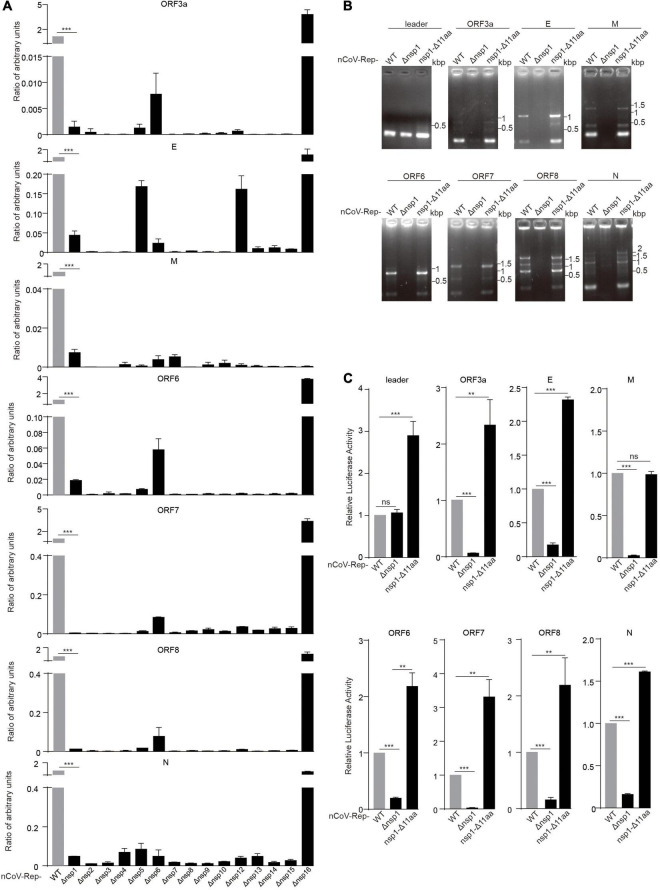
Nsp deletion impairs the replicative activity of the replicon. HEK293T cells were transfected with nCoV-Rep WT or nCoV-Rep-Δnsps and GFP, which was used as the control to normalize the transfection efficiency. 48 h post-transfection, the cells were harvested for qPCR assay with a 5-s extension time to quantify the subgenomic RNAs (Jin et al., 2021). The relative amounts of subgenomic RNAs for nCoV-Rep WT and Δnsp1-16 were depicted in **(A)**. Similarly, the relative amounts of subgenomic RNAs for nCoV-Rep WT, Δnsp1, and nsp1-Δ11 aa were analyzed with RT-PCR **(B)** with a 1-min extension time, which is enough to amplify all the possible subgenomic RNAs and qPCR **(C)**. Two-tailed unpaired Student’s *t*-test or one-way ANOVA with Bonferroni post-test correction was used to analyze the significance; **P < 0.01 and ***P < 0.001; ns, not significant.

To investigate the role of various nsps in viral replication and transcription, we transfected the WT replicon (nCoV-Rep) and the replicons with the deletion of various nsps (nCoV-Rep-Δnsp1 to 16) into HEK293T cells as described previously (Jin et al., 2021). We examined the subgenomic RNAs using quantitative PCR (qPCR) with the forward primer in the leader sequence and the reverse primers in 5’ UTR, ORF3a, E, M, ORF6, ORF7, ORF8, and N (Figure 3A). To only synthesize the products from the template composed of a direct fusion of leader sequence and coding regions of various ORFs, we reduced the extension time to 5 s as discussed previously (Jin et al., 2021). The results showed that except nsp16 deletion, the synthesis of each subgenomic RNA is largely impaired, indicating that all the nsps except nsp16 could play an indispensable role in the viral replication and transcription.

Deletions in nsp1 of SARS-CoV-2 variants were reported, indicating that nsp1 may not be essential for viral replication and transcription (Lin et al., 2021b). To clarify the different impacts on viral replication between the deletion of whole nsp1 and Δ500–532 in nsp1, we constructed the replicon with the reported deletion of 11 amino acids in nsp1 (nCoV-Rep-nsp1-Δ11 aa) (Lin et al., 2021b) and examined the synthesis of subgenomic RNAs. The results showed that unlike the replicon with the deletion of the whole nsp1 (nCoV-Rep-Δnsp1), nCoV-Rep-nsp1-Δ11 aa gave an increased synthesis of many subgenomic RNAs, in agreement with the previous report (Lin et al., 2021b; Figures 3B,C). This piece of data suggested that nsp1 plays an indispensable role in viral replication and transcription despite its unfavorable functions mentioned above.

### Non-structural Protein14 Reconstitution Can Rescue the Impaired Replication Caused by the Non-structural Protein14 Deletion

**FIGURE 4:**
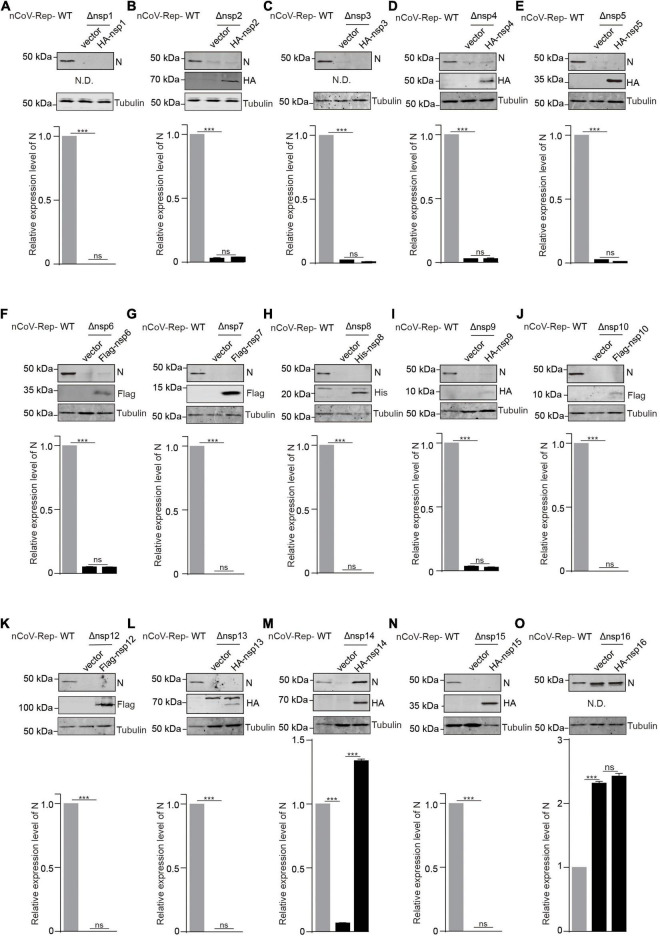
*In trans* expression of nsp14 rescues the nsp14-deletion-induced impaired replication. **(A–O)** HEK293T cells were transfected with nCoV-Rep WT or nCoV-Rep-Δnsp with vector or corresponding nsp expressing plasmid. 48 h post-transfection, the cells were collected and subjected to WB, blotted with indicated antibodies. N is the nucleocapsid protein antibody. N.D. is not detected. The densitometry of immunoblot bands was determined using Image Studio™ Lite Software (LI-COR Biosciences). The amounts of N protein were normalized with tubulin. Two-tailed unpaired Student’s *t*-test was used to analyze the significance; ***P < 0.001; ns, not significant.

Next, we asked whether the expression of non-replicon nsps *in trans* could rescue the impaired replication caused by the nsp deletion. We cotransfected nCoV-Rep-Δnsp1 to 16 with nsp1 to 16 expressing plasmid into HEK293T cells. Besides quantitative PCR, we examined the N protein expression using Western blotting (WB). In agreement with the qPCR result, except nsp16, the nsp deletions largely impair N protein expression (Figure 4 and Supplementary Figure 3). We found that the nsp14 could rescue the impaired viral replication caused by the nsp14 deletion, as indicated by the significantly increased expression of N protein by nsp14 expression compared with vector control. The nsp16 expression *in trans* could not increase the N expression, which is not affected by the nsp16 deletion, further supporting a dispensable role of nsp16 in viral replication and transcription (Figure 4 and Supplementary Figure 3). Since we failed to detect the expression of nsp1, nsp3, and nsp16, whether these nsps could rescue the impaired viral replication by corresponding nsp deletion is still not determined.

## Discussion

It is well recognized that nsps derived from the replicase polyprotein of SARS-CoV-2 play crucial roles in viral replication and transcription. However, whether all these nsps are indispensable for viral replication and transcription is still not fully defined. Here, we constructed the replicon of SARS-CoV-2 with the deletion of each nsp and verified the replication activity of the mutant replicons. We found that except for nsp16, all the deletions of nsp impaired the replication of the viral replicon (Figure 3A). The effect caused by the deletion of nsps should not be due to the low cleavage efficiency between the two nsps adjacent to the deleted nsp, because the cleavages on the reconstructed cleavage sites are verified with our 3CLpro/PLpro activity reporter system (Figure 1). By restoring the nsp14 expression, the nsp14 deleted replicon regained the replication activity and gave the expression of N gene at the 3’ terminal of viral genome (Figure 4).

In general, our findings suggest that the viral replication reliances on each nsp are varied. We found that the replicon with the deletion of nsp16 is still able to replicate itself and gives the expression of N gene, which is dependent on the discontinuous mechanism (Hussain et al., 2005). This piece of data indicating the viral 2’-O-methylation by nsp16 is likely dispensable for viral replication. Nsp16 could help the viral RNA escape from the recognition of the host innate immune system by decorating the viral RNA with 2’-O-methylation (Zust et al., 2011). We noticed an apparent increase in N gene expression, indicating that the deletion of nsp16 may promote translation efficiency. Indeed, the previous studies (Hoernes et al., 2016; Choi et al., 2018) uncovered that 2’-O-methylation decreases translation efficiency by disrupting tRNA decoding during translation elongation. Thus, we infer that besides facilitating the escape from the host immune monitoring, nsp16 could tune the expression of various subgenomic RNAs.

We found that nsp14 could rescue the replication of nsp14-deleted replicon, but the other nsps could not, indicating that nsp14 could function as a separate protein, not tightly associated with RTC as other nsps. Considering that the two reactions mediated by 3’-5’ exoribonuclease (ExoN) and N7-guanine methyltransferase (N7-MTase) activities of nsp14 could perform independently, we infer that hampering the association of nsp14 with viral RTC may not be a feasible antiviral strategy.

The expression of well-known replication-associated proteins, including nsp7, nsp8, nsp9, nsp10, nsp12, nsp13, and nsp15, could not recover the impaired replication of the viral replicon with the corresponding nsp deletion, indicating that the viral RTC assembly likely prefers the *in cis* component nsps generated from the same original polyproteins. The preference is likely due to the time window and the subcellular location for the viral RTC assembly (van Hemert et al., 2008; Acheampong et al., 2022). The exogenous expression of some nsps cannot fully satisfy the requirements, so these nsps cannot restore the assembly and the proper function of viral RTC.

Three nsps containing transmembrane domains, nsp3, nsp4, and nsp6, exhibited indispensable functions in the viral replication, indicating that the membrane structure or the location of RTCs could be essential for the efficient replication of viral RNAs by providing an essential microenvironment (Cortese et al., 2020). Mutations on the cleavage sites of nsp7 to nsp10 were reported to have a different impact on the viral replication compared with the in-frame deletions of nsp7 to nsp10 coding sequences. The mutation on the nsp9-nsp10 cleavage site only resulted in an attenuated viral replication, while the deletion of the coding sequence of nsp7-nsp10 was lethal for mutant viruses, consistent with our study (Deming et al., 2007). Although no replication-associated function of nsp2 is uncovered, the deletion of nsp2 largely impaired the replication of viral replicon, consistent with the previous reports that the titers of progeny virus by SARSΔnsp2 infection were ∼1-log_10_ reduced compared to wild-type infections (Graham et al., 2005), indicating that investigation on the nsp2’s replication-associated function is a potential research direction.

We acknowledge that our findings only briefly described the role of each nsp on viral replication and transcription. The conclusion drawn here based on the replicon system awaits further studies in detail using different systems, such as recombinant live viruses. Our findings here may provide a new angle to look at the role of various nsps in viral replication, suggesting more essential nsps and associations between nsps for antiviral strategy development.

## Materials and Methods

### Cell Culture and Transfection

HEK293T cells were cultured in Dulbecco’s Modified Eagle’s Medium (DMEM) (Thermo Fisher Scientific, C11960500), supplemented with 10% fetal bovine serum (Thermo Fisher Scientific, A3160801), 100 unit/mL penicillin, and 100 μg/mL streptomycin (Thermo Fisher Scientific, 15140-122) at 37°C with 5% CO_2_.

HEK293T cells were transfected at approximately 60% confluency with various nCoV-Rep vectors using Hieff Trans™ Liposomal Transfection Reagent (Yeasen Biotech, Cat#40802ES03) following the manufacturer’s instructions. 48 h post-transfection, the cells were subjected to various assays.

### Plasmid Construction

The construction of Ub4-nsp1/3-FL to Ub4-nsp14/16-FL is described as follows: First, the oligos of the positive and negative strands (1 μL for each at the concentration of 10 μM, listed in Table 1) were phosphorylated with T4 polynucleotide kinase (NEB M0201S) in T4 ligase buffer (NEB M0202S) at 37°C for 30 min. Second, the oligos were denatured for 10 min at 95°C and cooled down slowly (approximately 30 min) to room temperature. Third, the Ub4-FL (Du et al., 2021) was linearized with *Hin*dIII and purified. Lastly, the annealed oligos were ligated with linearized Ub4-FL at the ratio of 1:10, and the ligation products were transformed into the DH5α component cells. The clones were verified with Sanger DNA sequencing (Tsingke Biotechnology Co., Ltd., Beijing).

**TABLE 1:** Primers for cloning cleavage sites into Ub4-FL vector.

Primer name	Forward primer (5′–3′)	Reverse primer (5′–3′)
*Hin*dIII*-*Δnsp2	AGCTTCGTGAGCTTAACGGAGGGGCACCAACAAAGGTTA	AGCTTAACCTTTGTTGGTGCCCCTCCGTTAAGCTCACGA
*Hin*dIII*-*Δnsp3	AGCTTTTCACACTCAAAGGCGGTAAAATTGTTAATAATA	AGCTTATTATTAACAATTTTACCGCCTTTGAGTGTGAAA
*Hin*dIII*-*Δnsp4	AGCTTAATGTGGCAACTTTACAAAGTGGTTTTAGAAAAA	AGCTTTTTTCTAAAACCACTTTGTAAAGTTGCCACATTA
*Hin*dIII*-*Δnsp5	AGCTTACCTCAGCTGTTTTGCAGAGTGCAGTGAAAAGAA	AGCTTTCTTTTCACTGCACTCTGCAAAACAGCTGAGGTA
*Hin*dIII*-*Δnsp6	AGCTTTCAGGTGTTACTTTCCAATCTAAAATGTCAGATA	AGCTTATCTGACATTTTAGATTGGAAAGTAACACCTGAA
*Hin*dIII*-*Δnsp7	AGCTTAAAGTAGCCACTGTACAGGCTATAGCCTCAGAGA	AGCTTCTCTGAGGCTATAGCCTGTACAGTGGCTACTTTA
*Hin*dIII*-*Δnsp8	AGCTTAACAGGGCAACCTTACAAAATAATGAGCTTAGTA	AGCTTACTAAGCTCATTATTTTGTAAGGTTGCCCTGTTA
*Hin*dIII*-*Δnsp9	AGCTTTCTGCTGTCAAATTACAGGCTGGTAATGCAACAA	AGCTTTGTTGCATTACCAGCCTGTAATTTGACAGCAGAA
*Hin*dIII*-*Δnsp10	AGCTTGCCACAGTACGTCTACAATCAGCTGATGCACAAA	AGCTTTTGTGCATCAGCTGATTGTAGACGTACTGTGGCA
*Hin*dIII*-*Δnsp12	AGCTTCGCGAACCCATGCTTCAGGCTGTTGGGGCTTGTA	AGCTTACAAGCCCCAACAGCCTGAAGCATGGGTTCGCGA
*Hin*dIII*-*Δnsp13	AGCTTCCGCATACAGTCTTACAGGCTGAAAATGTAACAA	AGCTTTGTTACATTTTCAGCCTGTAAGACTGTATGCGGA
*Hin*dIII*-*Δnsp14	AGCTTAATGTGGCAACTTTACAAAGTTTAGAAAATGTGA	AGCTTCACATTTTCTAAACTTTGTAAAGTTGCCACATTA
*Hin*dIII-Δnsp15	AGCTTACTTTTACAAGACTTCAGTCTAGTCAAGCGTGGA	AGCTTCCACGCTTGACTAGACTGAAGTCTTGTAAAAGTA

The nCoV-Rep was constructed in our previous work (Jin et al., 2021). Considering the vectors’ capacity in cloning and ability to stably maintain the foreign DNA fragments, we employed the bacterial artificial chromosome (BAC) vector to clone the full-length cDNA of SARS-CoV-2. So far, six strategies have been successfully applied to construct the reverse genetics systems of coronaviruses: the RNA recombination-based, the vaccinia virus vector-based, the yeast-based recombination system-based, the circular polymerase extension reaction-based, BAC vector-based, and the *in vitro* ligation-based strategies. Among these strategies, BAC-based and the *in vitro* ligation-based strategies are the most widely used to construct reverse genetics systems of coronaviruses (Wang et al., 2022). Compared with the *in vitro* ligation-based strategy, the BAC-based strategy is more component in constructing the biosafe replicon and performing no live virus-involved quantitative studies on the replication and transcription of viral RNAs. To achieve the expression of viral genomic cDNA sequence in cells, we fused type II promoter CMV with the N-terminus of viral genomic cDNA and installed a transcriptional terminator BGH downstream of the C-terminus of viral genomic cDNA. To obtain the authentic 3′ terminus of viral genomic RNA, we inserted the HDV ribozyme between 3’ terminus of viral RNA and the BGH terminator. After the viral RNA is transcribed by the CMV promoter, the RNA sequence derived from BGH terminator can be removed through HDV ribozyme-mediated splicing mechanism, and the complete viral RNA is generated.

The constructions of nCoV-Rep-Δnsp1 to nCoV-Rep-Δnsp15 are described as follows. First, two unique restriction sites upstream and downstream of the nsp to be removed in nCoV-Rep (Jin et al., 2021) were selected. The middle forward/reverse primers (Table 2) include 3’ terminal sequence of the nsp prior to the nsp to be removed, 5’ terminal sequence of the nsp after the nsp to be removed, and the sequence generating the cleavage sites for PLpro or 3CLpro. The fragments upstream and downstream of the nsp to be moved were amplified with the primer combinations of the primer upstream of the restriction site and middle reverse primer and that of the primer downstream of the restriction site and middle forward primer, respectively. The two fragments were assembled seamlessly with NEBuilder HiFi DNA Assembly Master Mix (NEB #E2621). The assembled new fragments were amplified with the primers upstream and downstream of the restriction sites and inserted into nCoV-Rep vector to replace WT fragment between the selected two restriction sites. The DH10B component cells were employed for transformation, and the clones were verified with Sanger DNA sequencing (Tsingke Biotechnology Co., Ltd., Beijing).

**TABLE 2:** Primers for constructing the fragments with the deletion of each nsp.

Primer name	Forward primer (5′–3′)	Reverse primer (5′–3′)
Δnsp1	GGTAAGATGGCATACACTCGCTATGTCGATAACAACTTC	CATAGCGAGTGTATGCCATCTTACCTTTCGGTCACACCCG
Δnsp2	GCTTAACGGAGGGGCACCAACAAAGGTTACTTTTGGTG	GTTGGTGCCCCTCCGTTAAGCTCACGC
Δnsp3	CGGTAAAATTGTTAATAATTGGTTGAAGCAGTTAATTAAAGTTAC	CCAATTATTAACAATTTTACCGCCTTTGAGTGTGAAGG
Δnsp4	CAAAGAATGTGGCAACTTTACAAAGTGGTTTTAGAAAAATGGCATTCCC	GTAAAGTTGCCACATTCTTTGTTGTTACAACATTAACAACTTGTCTAGTAG
Δnsp5	CAGCTGTTTTGCAGAGTGCAGTGAAAAGAACAATCAAGGG	CACTGCACTCTGCAAAACAGCTGAGGTGATAGAG
Δnsp6	GTTACTTTCCAATCTAAAATGTCAGATGTAAAGTGCACATCAGTAG	CTGACATTTTAGATTGGAAAGTAACACCTGAGCATTGTCTAAC
Δnsp7	GCCACTGTACAGGCTATAGCCTCAGAGTTTAGTTCCCTTC	CTGAGGCTATAGCCTGTACAGTGGCTACTTTGATACAAGG
Δnsp8	GGGCAACCTTACAAAATAATGAGCTTAGTCCTGTTGCACTAC	GCTCATTATTTTGTAAGGTTGCCCTGTTGTCCAG
Δnsp9	GTCAAATTACAGGCTGGTAATGCAACAGAAGTGCC	CATTACCAGCCTGTAATTTGACAGCAGAATTGGCCC
Δnsp10	GTACGTCTACAATCAGCTGATGCACAATCGTTTTTAAACG	GTGCATCAGCTGATTGTAGACGTACTGTGGCAGCTAAAC
Δnsp12	CCATGCTTCAGGCTGTTGGGGCTTGTGTTCTTTG	CAAGCCCCAACAGCCTGAAGCATGGGTTCGCG
Δnsp13	CAGTCTTACAGGCTGAAAATGTAACAGGACTCTTTAAAGATTGTAG	GTTACATTTTCAGCCTGTAAGACTGTATGCGGTGTGTACATAG
Δnsp14	CTTTACAAAGTTTAGAAAATGTGGCTTTTAATGTTGTAAATAAGG	CATTTTCTAAACTTTGTAAAGTTGCCACATTCCTACGTG
Δnsp15	CAAGACTTCAGTCTAGTCAAGCGTGGCAACCG	CGCTTGACTAGACTGAAGTCTTGTAAAAGTGTTCCAGAGG
Δnsp16	CCCAAAATTACAATAAACGAACAATCCGCGGGGC	GATTGTTCGTTTATTGTAATTTTGGGTAAAATGTTTCTACATGGCC
nsp1-Δ11aa	GTTCGGATGCTCGAACTGCAGAACTCGAAGGCATTCAGTACGG	GCCTTCGAGTTCTGCAGTTCGAGCATCCGAAC

The strategy to construct nCoV-Rep-nsp1-Δ11aa was described previously for introducing mutations in nsp12 and nsp14 (Jin et al., 2021). In brief, the segment between *Bsi*WI and *Nhe*I was chosen for mutagenesis. The forward and reverse primers (Table 2) for generating the deletion of 500–532 nt (Δ11aa) were used to amplify the fragments upstream or downstream of the mutant site. The two fragments were assembled seamlessly with NEBuilder HiFi DNA Assembly Master Mix. The assembled products were amplified by PCR and used to replace the wild-type *Bsi*WI-*Nhe*I segment.

The construction of nCoV-Rep-Δnsp16 is relatively straightforward compared with other nsp deletion mutants. The *Axy*I-*Sac*II fragment without nsp16 was inserted into nCoV-Rep vector to replace WT fragment between *Axy*I and *Sac*II sites.

The construction of LPC-nsp1 to LPC-nsp16 is referred to as in our previous work (Jiang et al., 2021).

### Dual-Luciferase Reporter Assay

One day prior to transfection, HEK293T cells (1 × 10^5^) were plated in 24-well plates. Various Ub4-CS-FL (0.5 μg), RL-TK (0.1 μg), and LPC-3CLpro-HA or LPC-PLpro-HA were transfected into the cells using Hieff Trans™ Liposomal Transfection Reagent. Forty eight hour post-transfection, the cells were lysed in 50 μL 1x Passive Lysis Buffer (PLB, Promega, E1941). The activities of firefly and renilla luciferase were measured using Dual-Glo^®^ Luciferase Assay System to determine the relative luciferase activities.

### RNA Extraction, Real-Time Quantitative PCR, and RT-PCR

Total RNA was isolated from the HEK293T cells using TRIzol reagent (Thermo Fisher Scientific, Cat#15596026) and treated with RNase-free DNase (Takara, Dalian, China, EN0521) according to the manufacturer’s instructions. The quality of RNA was examined using the electrophoresis on 1% agarose gel, and the purity of RNA was verified on the basis of the ratio of OD260/280 on NanoDrop One (Thermo Scientific). Two μg of total RNA was used as a template to synthesize cDNA using the cDNA synthesis kit (Thermo Fisher Scientific, Cat#K1622). The cDNAs were subjected to the real-time quantitative PCR (qPCR), which were performed on a 96™ Real Time PCR Detection System (Applied Biosystems™ 7500), in 10 μL reaction mixtures containing 5 μL SYBR^®^ TB Green^®^ Premix Ex Taq™ (Tli RNaseH Plus) (Takara, Dalian, China, Cat#RR420A). The thermal profile consists of 30 s at 95°C, followed by 40 cycles of 5 s at 95°C and 5 s at 60°C. The 2^–ΔΔCT^ method was used to calculate the relative gene expression values. The details for the design of primers and qPCR conditions were described as previously (Jin et al., 2021). The RT-PCR was performed with 2 × Hieff^®^ PCR Master Mix (Yeasen, Shanghai, China, 10102ES08), extension time of PCR program is 1 min, and PCR products were examined in the 1.5% agarose gel after DNA electrophoresis.

### Western Blot

Cell samples were collected and lysed in RIPA lysis buffer (150 mM NaCl, 1% NP-40, 0.1% SDS, 1 mM EDTA, and 50 mM Tris-HCl pH7.4, supplemented with cOmplete™ Protease Inhibitor Cocktail). All samples’ concentration was quantified with BCA assay (Thermo Fisher Scientific, 23227) following the manufacturer’s instructions. The lysates were subjected to SDS-PAGE and then transferred to nitrocellulose membranes. The membranes were incubated in PBS containing 0.05% Tween 20 and 5% non-fat milk (blocking buffer) for 1 h at room temperature and then left in the blocking buffer containing primary antibody of HA (Proteintech, 10011878), His (Proteintech, 10004365), Flag (Proteintech, 00098867), Tubulin (Proteintech, 66031-1-Ig), or Nucleocapsid (Sino Biological, 40143-R019) at 4°C overnight. The following day, after being washed with PBS containing 0.05% Tween 20, the membranes were incubated with appropriate secondary antibody (Goat anti-Rabbit, LI-COR, D10121-05; Goat anti-Mouse, LI-COR, D10217-05, at 1:10,000). The final blots were developed on Odyssey CLx Imaging System (Li-COR Biosciences).

### Statistics

Except for specially stated, all the experiments were performed at least three times. The data analyses were finished using Student’s *t*-test of SPSS (version 21.0; SPSS Inc., Chicago, IL). When *P*-value was less than 0.05, the results were considered significant.

## Data Availability Statement

The original contributions presented in the study are included in the article/Supplementary Material, further inquiries can be directed to the corresponding author/s.

## Author Contributions

XP and J-AP conceived the ideas, designed the experiments, and wrote the manuscript. All authors performed experiments or data analysis, read, and approved the final manuscript.

## Conflict of Interest

The authors declare that the research was conducted in the absence of any commercial or financial relationships that could be construed as a potential conflict of interest.

## Publisher’s Note

All claims expressed in this article are solely those of the authors and do not necessarily represent those of their affiliated organizations, or those of the publisher, the editors and the reviewers. Any product that may be evaluated in this article, or claim that may be made by its manufacturer, is not guaranteed or endorsed by the publisher.

## Supplementary Material

Supplementary Figure 1 Design of each nsp deletion. Each nsp was deleted by fusing its adjacent upstream and adjacent downstream nsps, except nsp1 and nsp16. The new cleavage site sequences were depicted. The deletion of nsp1 and nsp16 left a new start codon before nsp2 and a stop codon after nsp15, respectively.

Supplementary Figure 2 Sanger sequencing results for nCoV-Rep-Δnsps. The Sanger sequencing results covering the newly formed cleavage site regions in various nCoV-Rep-Δnsps were depicted.

Supplementary Figure 3 Investigation of *in trans*-rescue function of various nsps in the viral replication by quantifying the amount the N (related to Figure 4). **(A–O)** The RNA samples described in Figures 4A–O were subjected to real-time quantitative PCR assay. The amounts of N subgenomic RNA (sgRNA) from various samples were normalized with internal control GAPDH.
