# Supplementary figures and images for: Genome-Wide Analysis of the Indispensable Role of Non-structural Proteins in the Replication of SARS-CoV-2
Source: Front Microbiol. 2022 Jun 1;13:907422. doi: 10.3389/fmicb.2022.907422 (PMC9198553; doi:10.3389/fmicb.2022.907422)

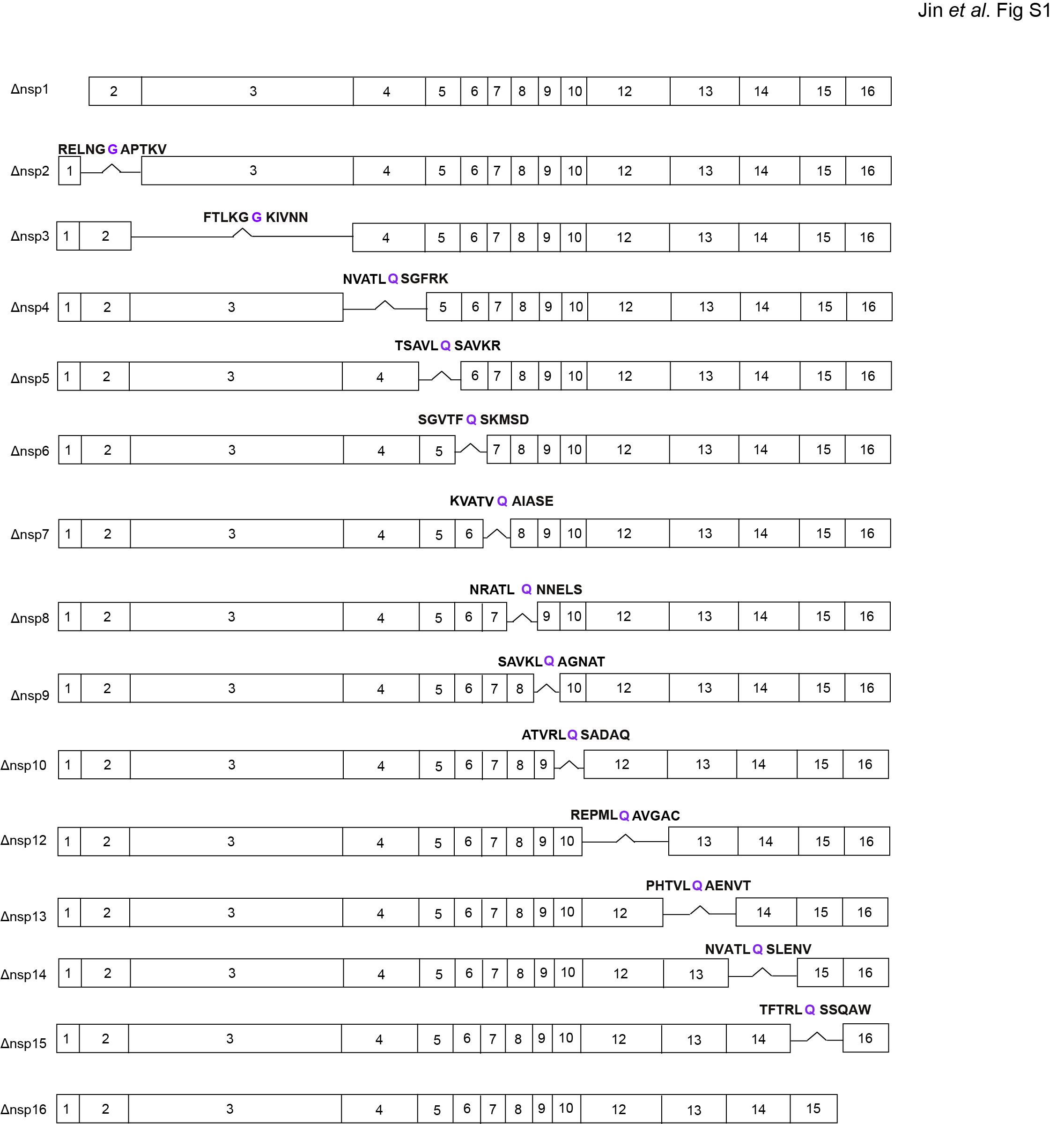

Supplement: Supplementary Figure 1 — Design of each nsp deletion. Each nsp was deleted by fusing its adjacent upstream and adjacent downstream nsps, except nsp1 and nsp16. The new cleavage site sequences were depicted. The deletion of nsp1 and nsp16 left a new start codon before nsp2 and a stop codon after nsp15, respectively. [file Image_1.JPEG]

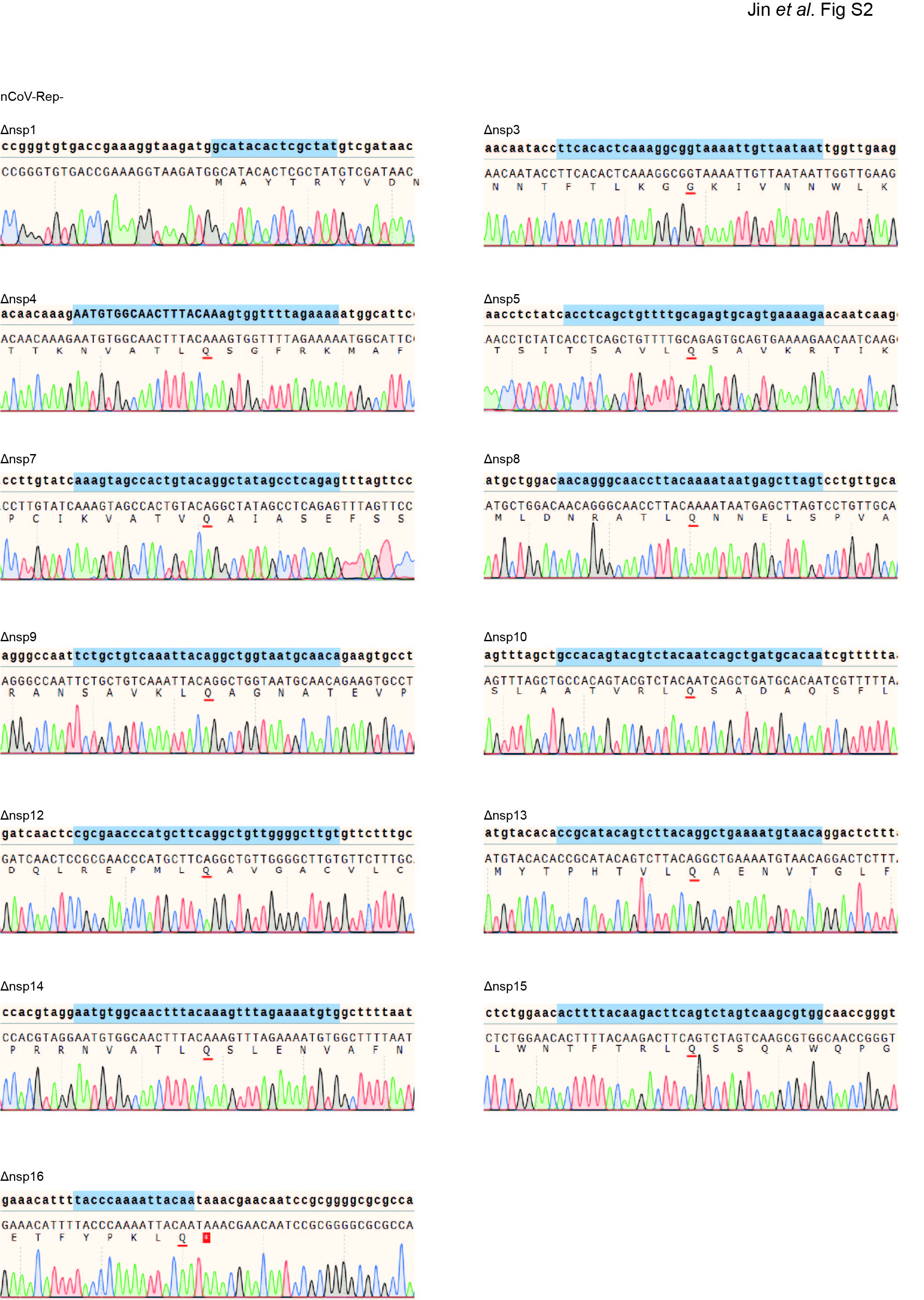

Supplement: Supplementary Figure 2 — Sanger sequencing results for nCoV-Rep-Δnsps. The Sanger sequencing results covering the newly formed cleavage site regions in various nCoV-Rep-Δnsps were depicted. [file Image_2.JPEG]

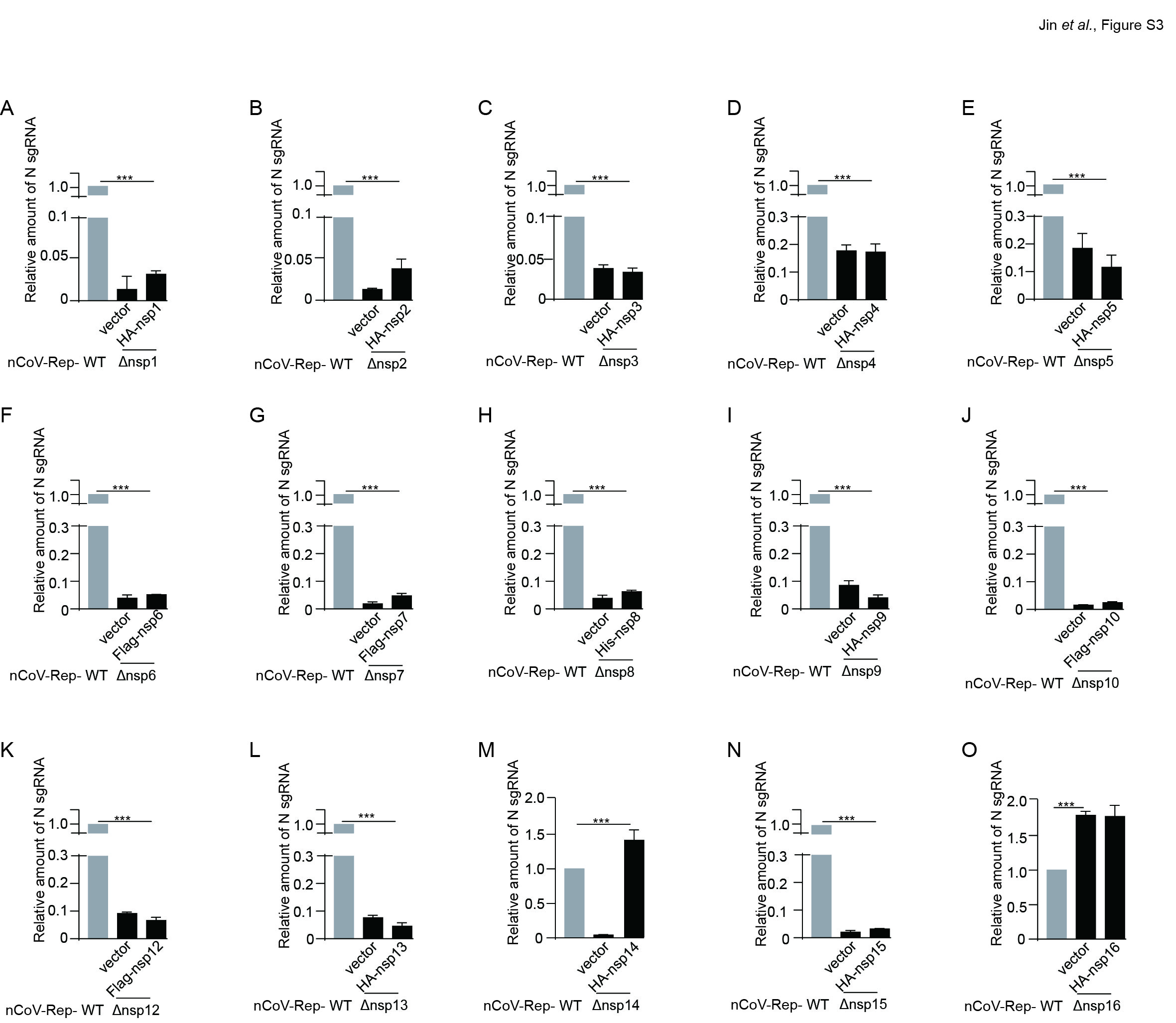

Supplement: Supplementary Figure 3 — Investigation of in trans-rescue function of various nsps in the viral replication by quantifying the amount the N (related to Figure 4). (A–O) The RNA samples described in Figures 4A–O were subjected to real-time quantitative PCR assay. The amounts of N subgenomic RNA (sgRNA) from various samples were normalized with internal control GAPDH. [file Image_3.JPEG]
